# Acceptability of the LetSync App Wireframes for an mHealth Intervention to Improve HIV Care Engagement and Treatment Among Black Partnered Sexual Minority Men: Findings from In-Depth Qualitative Interviews

**DOI:** 10.2196/43676

**Published:** 2023-08-25

**Authors:** Nozipho Becker, Hyunjin C Kim, Darius J Bright, Robert Williams III, Joaquin A Anguera, Emily A Arnold, Parya Saberi, Torsten B Neilands, Lance M Pollack, Judy Y Tan

**Affiliations:** 1 Office for Inclusive Excellence Colorado State University Fort Collins, CO United States; 2 Center for AIDS Prevention Studies Division of Prevention Science, Department of Medicine University of California San Francisco San Francisco, CA United States; 3 Neuroscape Department of Neurology University of California San Francisco San Francisco, CA United States

**Keywords:** digital health, mobile health, mHealth, mobile app, app, Black sexual minority men, couples, HIV care engagement, HIV treatment, United States, mobile phone

## Abstract

**Background:**

HIV disparities continue to be a significant challenge affecting Black sexual minority men in the United States. Inadequate engagement and retention of patients in HIV care has been associated with poor health outcomes. Interventions to improve sustained commitment to HIV care are needed. Mobile health interventions can help facilitate access to and use of HIV health services, particularly among individuals at risk for disengaging with care.

**Objective:**

We designed the *LetSync* app wireframes for a mobile health intervention using a couple-centered design approach to improve HIV engagement and treatment among Black sexual minority men and their partners. The objective of this study was to gauge future app user interest and elicit feedback to improve the design, development, and usability of the *LetSync* app.

**Methods:**

We conducted in-depth interviews with 24 Black sexual minority men to assess the acceptability of the *LetSync* app wireframes between May 2020 and January 2021. Participants reviewed the *LetSync* app wireframes and provided feedback regarding perceived usefulness and interest in future app use and suggestions for improvement.

**Results:**

Participants indicated interest in the future *LetSync* app and noted that the wireframes’ features were acceptable and usable. In our study, the future *LetSync* app was frequently referred to as a potential resource that could help facilitate users’ engagement in HIV care through the following mechanisms: enable scheduling of appointments and timely reminders for clinic visits; help improve HIV medication adherence; encourage and motivate participants to ask questions to their health care provider and stay engaged in conversations during clinic visits; facilitate effective communication by assisting couples with planning, coordination, and management of daily routines; help participants understand their partner’s health needs, including access to and use of health care services; and facilitate participants’ ability to improve their relationship skills, partner support, and self-efficacy in managing conflict. In addition to near-universal interest in potential daily app use, study participants indicted that they would recommend the *LetSync* app to other family members, friends, and people in their social networks who are living with HIV.

**Conclusions:**

Our findings revealed considerable interest in future app use for HIV care management, which could possibly increase the chance of the *LetSync* app being successfully adopted by Black sexual minority men in couples. Owing to its interactive and couple-centered approach, the *LetSync* app could help improve communication between Black sexual minority men and their partners and health providers. In addition, the *LetSync* app could provide an acceptable modality for these men to receive support in accessing HIV care services.

## Introduction

### Background

HIV disparities continue to be a significant challenge for Black people in the United States, particularly among Black gay, bisexual, and other men who have sex with men. In 2019, Black men accounted for 68% of the HIV incidence, with over 80% of new infections attributed to sexual minority men [1]. For Black sexual minority men, the substantial disparities in HIV can be seen through the intersection of inequities resulting from discrimination based on sexual orientation and ethnicity or racial background, especially as it pertains to access to and use of HIV care services. In 2019, approximately 59% of Black sexual minority men were retained in care at some point, and approximately one-fourth of them missed at least one medical appointment [2]. Declining retention rates pertaining to HIV care are disconcerting, as they suggest lapses in the access to and use of HIV prevention and treatment services in this population.

### Partner Support and HIV Care Engagement

Engagement and retention of patients in HIV care is the most significant component of therapy necessary to decrease HIV transmission and achieve viral suppression and is crucial for optimal HIV health management [3]. Among other key factors, social and partner support has been shown to be associated with improved HIV health outcomes among sexual minority men [4]. In a study investigating couple-level dynamics and multilevel challenges among Black sexual minority men, participants described HIV care as a collaborative process, which they felt was most effective when partners coordinated with each other to solve problems they faced regarding access to and use of HIV care services [5]. In another study that examined dyadic coordination regarding access to HIV care, although there were variations in preference regarding the extent of involvement, partner involvement was reported as playing a significant role in facilitating HIV care engagement and treatment among Black sexual minority male couples [6]. As part of our formative work, we conducted a study investigating mobile technology use among older Black sexual minority men living with HIV, in which participants showed considerable interest toward mobile technology, and several reported having used mobile technology interventions to engage in HIV care services [7]. This suggests that mobile health (mHealth) interventions could play a supportive role in improving self-care and health outcomes for Black sexual minority men living with HIV, particularly for those at risk for disengaging from care.

### The Role of mHealth in HIV Prevention and Treatment

Forgetfulness and lack of family support have been reported as significant barriers to treatment adherence and HIV care engagement [8]. In a study conducted among sexual minority men in the United States, forgetfulness was found to be a significant factor associated with missed treatment doses and clinic visits among participants [9]. As various features of the *LetSync* app intervention (*digital pill case* and *appointment minder)* would allow app users to set up reminders to take their daily doses, attend clinic visits, and pick up their medication refills, the *LetSync* app intervention could help improve antiretroviral therapy treatment adherence and HIV care engagement. In addition, the *my action plan* feature would be instrumental in facilitating effective communication by assisting couples with the planning, coordination, and management of physician or clinic appointments and couples’ daily routines. Findings from these studies demonstrate a need for developing mHealth interventions focused on improving HIV care engagement and treatment adherence for partnered Black sexual minority men. In recent years, the tremendous increase in access to and use of mobile technology in the United States has been directly correlated with increased innovation in health technologies, including the development of mHealth interventions for chronic disease management. Currently, mHealth interventions are increasingly being used to improve health outcomes across the HIV care continuum and have been shown to facilitate HIV prevention (increased HIV testing, condom use, and sexual health) [10,11] and improve HIV medication adherence [12]. In a recent review of studies of Black sexual minority men living with HIV in the United States, mHealth interventions were significantly associated with improvements in antiretroviral therapy adherence and viral suppression among participants [13].

### Study Justification and Objective

Despite the emergence of mHealth and studies of lifestyle behavior changes, very few mHealth interventions have developed a mobile app to address HIV care engagement and treatment adherence among Black sexual minority men. So far, most HIV-related mHealth interventions focused mainly on either electronic and web-based interventions or one-way messaging such as SMS text messages, emails, or surveys [13,14]. SMS text messages are generally shown to be effective in improving medication adherence across a variety of populations and settings. However, some studies have shown that combining SMS text messages with other features (social media contact and app-based self-monitoring—all features that are possible within the architecture of an app) shows promise for increasing the effectiveness of health interventions [15].

Although mobile-based HIV interventions have been developed for Black sexual minority men, most of the interventions developed so far have targeted individual users (single individuals rather than individuals in couples). To the best of our knowledge, this is the first study to develop a mobile app intervention aimed at improving HIV care engagement among Black sexual minority male couples. We designed the *LetSync* app wireframes for an mHealth intervention using a couple-centered design approach to improve HIV engagement and treatment among Black sexual minority men and their partners. The objective of this study was to gauge future app user interest and elicit feedback to improve the design, development, and usability of the *LetSync* app. Findings will be used to inform the design and development of a dyadic mHealth app intervention aimed at improving HIV care and treatment among Black sexual minority men in couples.

## Methods

### Overview

On the basis of formative studies [5-7], we designed the *LetSync* app wireframes for an mHealth intervention to improve HIV engagement and treatment among Black sexual minority men. We assessed the acceptability of the *LetSync* app wireframes among Black sexual minority men. Participants were recruited from web-based platforms by trained personnel between May 2020 and January 2021. This study was part of a large project exploring the use of mobile technology to increase engagement in HIV care among Black sexual minority men in couples.

### Recruitment

Owing to the shelter-in-place mandate related to the COVID-19 pandemic, recruitment was primarily conducted via web-based platforms (including dating apps and social media). Purposive sampling was used to recruit participants into this study. Detailed information regarding web-based recruitment procedures is described elsewhere [16]. In addition, the research team used a snowball sampling technique (through word of mouth) and worked with local community-based organizations to recruit other qualifying couples to participate in the study. Potential participants were invited to a phone screening to determine eligibility. Participants were eligible if (1) one or both partners were Black or African American, observed as male at birth, sexual minority men, and living with HIV; (2) they were in a relationship with a primary partner for at least 2 months; (3) both had a personal mobile phone; and (4) they were aged 18 years and older.

### Data Collection

The *LetSync* app wireframe assessment was conducted using one-on-one, in-depth, semistructured interviews with 24 Black sexual minority men in couples, regarding topics that were related to intervention development and the unique health needs of partnered Black sexual minority men living with HIV. Interviews were conducted via teleconference and lasted for approximately 1 hour. The *LetSync* app wireframes were hosted on an Adobe XD website supported by InVisionApp Inc. Interview questions (Multimedia Appendix 1) pertaining to the *LetSync* app wireframes assessed user interest and gathered feedback regarding future app features, which included specific designs to support dyadic interactions among Black sexual minority male couples, as informed by previous studies [5,7]. During the interviews, participants were asked to provide their perceptions and reactions regarding the following aspects of the *LetSync* app wireframes features: engagement (interactivity, potential user interest, and recommendations), functionality (performance, navigation, and ease of use), esthetics (appearance, layout, and visual appeal), and information quality (content appropriateness and suggestions for improvement). The wireframe assessment portion of the interviews proceeded as follows:

1. First, the interviewer displayed the *LetSync* app wireframes via screen share on Zoom. Examples of the *LetSync* app wireframes are shown in Multimedia Appendix 2.
2. Next, the interviewer demonstrated how the various app features worked by showing the corresponding wireframes. App features included (1) *appointment minder*, which is designed to help users schedule physician’s appointments, set reminders, and create a digital copy of a physical appointment card; (2) *my action plan*, a feature that would allow users to identify health problems, set up goals, and devise strategies they can use to achieve each goal; and (3) *digital pill case*, a feature designed to enhance medication adherence by allowing users to track adherence to each medication, creating a visual log to let them know when they have taken their daily doses.
3. Participants were asked to provide feedback about each of the features shown to assess the acceptability and usefulness of the future app for HIV care management.
4. Finally, the interviewer concluded by asking the participant to reflect about their general thoughts about the app wireframes and to provide suggestions for improvement.

### Data Analysis

Interviews were recorded, transcribed verbatim by a professional service, and subsequently coded using Dedoose software (version 9.0.62; SocioCultural Research Consultants), a secure, web-based, qualitative analytic software that is ideal for team-based coding. Data were analyzed using both inductive and deductive codes, first relying on the semistructured interview guide to generate thematic areas of content that would need to be defined and rigorously coded. This process was also augmented by inductive coding, where open coding was used to capture emergent themes and, later, define them. First, 4 members of the research team (HCK, DJB, EAA, and RW) independently reviewed the transcripts line by line to identify thematic content to be captured in our coding scheme and then used deductive and inductive codes to create a codebook, defining each code. The codebook was then applied to 1 pair of interview transcripts, with the entire team working together to discuss and apply the codes. Discrepancies were resolved through group consensus, and codes were revised as necessary. Once all members of the team felt confident to consistently apply the codes, interview transcripts were assigned to a primary coder and a secondary coder, who reviewed the coding decisions of the primary coder. Divergent opinions around code applications between the primary and secondary coders were discussed and resolved at regular analysis meetings of the large team. All transcripts were coded using Dedoose. Coded excerpts were extracted from Dedoose and were reviewed for consistency by multiple team members.

### Ethics Approval and Informed Consent

This study received ethics approval from the institutional review board of University of California, San Francisco (institutional review board protocol 15-18042). Informed consent was obtained on the web via Qualtrics (Qualtrics International Inc) from all participants included in the study.

## Results

### Participants’ Demographic Characteristics

In total, 24 participants reviewed the *LetSync* app wireframes via Zoom or phone in 2021 (Table 1). Most participants in our study (21/24, 88%) identified as Black or African American, and three-fourths (18/24, 75%) of the couples were not married. Participants in our study sample had been in an intimate relationship for approximately 2 (IQR 0.54-16.88) years. Half (12/24, 50%) of the participants were middle-aged men, and approximately three-fourths (17/24, 71%) of the participants lived with their spouses or partner. Most participants (18/24, 75%) attended college, and all (24/24, 100%) had access to the internet.

**Table 1:** Participants’ demographic characteristics (N=24).

Demographics			Values
Time in relationship (years), median (IQR)			1.75 (0.54-16.88)
**Age group (years), n (%)**			
	<30	6 (25)	
	30-49	12 (50)	
	≥50	6 (25)	
**Educational level, n (%)**			
	Less than high school	1 (4)	
	High school degree or General Educational Development	5 (21)	
	At least some college	18 (75)	
**Income (US $), n (%)**			
	<10,000	8 (33)	
	10,000-49,000	7 (29)	
	50,000-99,000	7 (29)	
	≥100,000	2 (8)	
**Marital status, n (%)**			
	Not married	18 (75)	
	Married or partnered	6 (25)	
**Ethnicity, n (%)**			
	Black or African American	21 (88)	
	White	1 (4)	
	Mixed or multiracial	2 (8)	
**HIV status, n (%)**			
	Positive	17 (71)	
	Negative	7 (29)	
**Cohabiting, n (%)**			
	No	7 (29)	
	Yes	17 (71)	
**Internet access, n (%)**			
	Limited minutes or data	2 (8)	
	Unlimited minutes or data	22 (92)	

Overall, participants indicated interest in the future *LetSync* app and noted that the wireframes’ features were acceptable and usable. Participants thought that the app wireframes were intuitive and that the layout and imagery were appropriate. In this section, we have reported participants’ feedback about three main features of the *LetSync* app wireframes, including (1) *appointment minder*, (2) *digital pill case*, and (3) *my action plan*. In addition, we have also presented findings regarding the study participants’ interest in future app use and suggestions for improvements.

### Appointment Minder

#### Overview

Participants expressed interest in using the *appointment minder* feature for planning and health management in the future. In addition to ease of use, study participants liked this feature because they believed it would be extremely helpful for organizing activities and scheduling clinic visits. Participants appreciated the idea of having an appointment card that could be incorporated into their calendar schedules:

Yeah. That sounds good. I think that would help...probably help people that use the tool to make their appointments.

participant ID (PID) P3801

Participants preferred the *appointment minder* be interactive. In addition to the date and name of the provider, participants envisioned this feature as having a built-in function that would allow them to specify the type of appointment (eg, laboratory work, x-ray, and physical examination) to aid in preappointment preparations (eg, fasting). Knowing the type of appointment would be instrumental in helping them prepare and organize questions that they would like to ask their providers during visits:

Well, yes...on like maybe your appointment’s today. It’s for this. Remember to always ask questions about this. Have you asked your doctor like about...your results from your previous appointment? Or make sure the doctor goes over your previous like labs, or something like that.

PID P1201

Participants reported that they would like *appointment minder* to incorporate a comment section where, before their appointments, participants could write down notes and questions that they would like to ask their health providers:

You need to prepare yourself to ask the doctor anything you need...You should ask your doctor about medication, medication side effects, you should ask your doctor about, you know, minor aches and pains. You should ask your doctor if anything changes in your health, with regarding your body, you know. Cause you can go through a lot of changes taking HIV medicine.

PID P1501

Others felt that this feature would enable them to stay engaged in conversations and help them remember the things they needed to discuss with their providers during clinic visits. In addition, participants thought that the app could help them document their experiences regarding medication side effects or challenges they might encounter regarding access to and use of health care, potentially playing a significant role in improving care delivery:

By asking you if you’re experiencing this type of side effect. And then, if you do, then it would take you to another screen that would help you - that would remind you to ask your doctor about it, or would suggest a way to either slow the medicine down, or ask your doctor to stop the medicine, or something like that.

PID P2002

Participants also viewed the *appointment minder* feature as a potential resource to help improve transparency between couples. Together with *my partner*, the *appointment* *minder* feature could allow easy sharing of information and planning of activities, thus helping to prevent schedule-related conflicts and unnecessary arguments between couples:

So, “My Partner” would help, too, as well. It’s just overall understanding. Like, I think both of those—“Appointments” and “My Partner”—are two key sections just to help you get through the day and know more about your partner. So that unnecessary arguments or reactions don’t happen.

PID P3702

In addition, participants commented about the feature’s potential to help couples build trust and foster strong relationships. They felt that *appointment minder* would enable them to better understand their partners’ health needs and could help them formulate strategies to provide physical, mental, or emotional support to their partners, particularly during stressful situations:

Just so that I know, like, that we know those dates and times. Like, to me it’s important to know that, because [partner’s name] and mine’s behavior—or our attitudes—could change days leading up to an appointment, depending on what we’re going in for. You know, we could be nervous, or we might—I feel like that would help just so something comes out the wrong way, we kind of know it’s nothing personal. It’s just they’re nervous about their upcoming appointment.

PID P3702

#### Suggestions for Improvement

Participants stated that they would prefer the *appointment minder* feature to also include generic questions or standard prompts. They envisioned this feature as having built-in prompts or questions that were tailored to the participants’ health conditions, which they could use as a guide to organize or develop questions that they should ask their providers during clinic visits. They felt that they would find this feature even more helpful if it incorporated typical follow-up response questions that were tailored to treatment side effects, laboratory results, and specific medical procedures or health conditions:

Honestly, I think the suggestion things should be there. I think there should be like a little comment section. So, like, you know, they did have questions, like they can write it down at that appointment and it pops up above it, saying remember to ask your doctor about quotation - not quotation marks but print. You know what I’m trying to say? Instead of calling or, you know, or buzzer, buzzer, buzzer with the comment, you know, you wrote down prior the visit up under it.

PID P1801

### My Action Plan

#### Overview

Participants indicated that they would like to use *my action plan* in the future to set overall goals for health management. In addition to *my action plan*’s potential to facilitate organizing and planning, participants liked the feature’s ability to help them *prioritize* goals and activities. They liked the idea of being able to document plans for both short-term and long-term goals such as taking medication daily, calling the physician about medication side effects, and scheduling follow-up appointments with their providers:

Mostly for plans, because I don’t typically plan. I more so, just keeping it up here and then doing it. But you know, just to see it written up or whatever, being in front of you to see those things like that, even if it’s a month plan or a year plan, to see where you are now and to see where you’re going to be, to see that you have three or four things.

PID P2401

Participants also felt that this feature would be instrumental in helping them with responsibilities they needed to remember. They noted that the *my action plan* feature would help them set important reminders for topics they would like to discuss with their health providers during visits and would allow them to track progress regarding their goals for health management:

*It’s good. It’s a good well-organized plan. Oh, that’s real good. Especially, yeah, the fact that you can say, like, I’m most likely going to forget — most likely going to forgetto pack that for the trip. So, it’ll highlight that and make sure you don’t forget to pack your meds and stuff like that.* [PID P1201]

In addition, participants were enthusiastic about the goal-setting function of this feature. They liked the idea of being able to create a step-by-step action plan to remind them about the various small actions that they needed to follow to achieve their health goals in a timely manner. Some participants felt that this feature would help to organize activities, especially when preparing for long trips or traveling. They envisioned *my action plan* as being able to help them prioritize health-related responsibilities such as packing their medication, taking medication on time, and arranging for prescription refills at pharmacies near their destination:

I think it would be great, because a lot of times, when we are traveling, we kind of do forget to take our meds because we’re excited about the travel. And you would, depending on how long you’re going to be gone, or you may be down to your last three or four meds and they won’t carry you over for your trip. So, you kind of would want to get in touch with the doctor and have them send a prescription, or you may want to do the same thing and find out if there’s a CVS or pharmacy where you’re going, where you can pick them up while you’re on your trip. I’ve done that before.

PID P4402

Although some participants appreciated the feature’s potential to help with organizing activities and health care plans, they were less enthusiastic about using this feature in the future, as they felt that they could access similar information such as refilling prescriptions, appointment schedules, and health plans from other provider-based mHealth platforms such as *MyChart:*

Well, that’s actually, that seems like a pretty good work flow map. But a lot that was mentioned on there, I can go to MyChart and deal with that.

PID P4201

#### Suggestions for Improvement

Although participants reported interest in using this feature in the future, they suggested having the goal-setting feature in *my action plan* arranged in descending order, starting from least likely to remember to most likely. A participant explained this as follows:

Most likely to forget — like most likely to, “Set aside time to pack meds.” Yeah. So, it will put that at the top. Cause I’m most likely — I mean, I could say least likely — let me see what am I saying? “Set aside time to pack meds.” Less likely to do that...It might need to put what I’m least likely to do to make sure I’m reminded about that more.

PID P1201

Other participants suggested having additional questions incorporated within this feature to help them identify potential barriers that may inhibit their ability to address their health needs and prevent them from achieving their health management goals:

Okay. Yeah, I think when I’ve done planning before, on all the goals - one of the questions that I ask myself is, “What can get in the way of me doing this particular goal?” Because if it’s on the list, they’re all important. So I don’t get to pick the ones that I’m most likely to do, therefore that’s the most important. I think sometimes I have to step back and ask the question, “Why am I not doing this? It’s clearly important to my health. But this is the one that I have to - either I slip or I ignore it, or something.” I think it would be okay for the app to ask you, “What could get in your way of doing this?” Or, “What could you do to make this more likely that you would do this than less likely?” Something like that.

PID P5101

Participants also thought that *my action plan* could help motivate health management. They expressed the importance of setting achievable goals, so that participants would not be discouraged by lack of progress. A participant recommended adding a “reward system” (eg, colorful text or imagery) that would be activated when participants achieved their goals and benchmarks for health management:

My only thought is, like when there is a congratulations or achievement or something, good to read stuff, I would say probably I’d add imagery there. No direct imagery, but maybe like green text maybe. For like a positive...Because looking at this, we can’t really tell that, oh, there’s an achievement...The achievement, it may be a good idea to have...Or maybe like a gold color. Just maybe some kind of just separate imagery from a regular notification. That’s the only thing I would say.

PID P4602

### Digital Pill Case

#### Overview

Participants provided positive feedback regarding the *digital pill case* feature. They thought that *digital pill case*, including the color-coded before or after display buttons, would help monitor their daily medication doses and improve treatment adherence. Participants commented that they particularly liked the feature’s ability to use color-coded buttons as a reminder for when they have not taken their medication on time. They also liked the idea of the app creating a log after they have taken their daily doses and having their medication schedule incorporated into their calendars:

It would make sense to like make that more like - more like a pizza pie type shape. So, you can see the sections of the week being - being missed and fulfilled. Sort of...like a seven-slice pizza - like pie chart. That would show progress, I think. And it would show the missing days, if you didn’t check in. And like a really easy to digest digital pattern. In a visual way.

PID P2002

When asked whether they would prefer the app to send a follow-up message for a status update 30 minutes after taking their medication, participants stated that although this could be a good feature to have, they thought regular prompts might be somewhat cumbersome:

That would be good - it may be a little - it may be a little too much.

PID P2002

When asked whether they would like this feature to synchronize with their alarm or calendars and whether they would like having pop-up reminders that display over the top of the screen to notify them when it is time to take their medication, participants indicated that they would like that function and thought that it would be a very useful and effective reminder:

I feel like that’s a great idea. Because that is something, I know, in the past, I have ignored a reminder because it was just a push notification. But, if it covers the screen and it’s popping up over the app, then, yeah, it’s difficult to ignore.

PID P4601

Others commented that they would find this feature to be helpful for tracking daily medication doses and monitoring treatment adherence:

You know what I do? I look at the pill case and see, because it’s labeled Sunday through Saturday. And if it’s empty, then I know I took it.

PID P4701

#### Suggestions for Improvement

Some participants indicated that they would like a more refined visual display of *digital pill case.* They envisioned this feature as having the ability to accommodate all the applicable medications that were specific to each user’s individual and unique health conditions:

I think that’s good. Why not take it a step further? I mean, if you’re going to have a Digital Pill Case like that, why not even have—because one thing I noticed about [name of index] with his medication is, he has medications and they’re all different names for different medications. But he doesn’t know what his medication is for. So if you guys had those tabs, could they also, like, name it, say? “Oh, pain medication! Fever reducer.”

PID P0902

Other participants wanted this feature to allow them to personalize the types of medication according to individual health needs and preferences:

Yeah, something they can have whatever personal thing they want to add to the side. So like when you got the tab in there, and like this is their HIV medication they could instead of “HIV medication” they could put “Every Day Life Pill,” or something like that.

PID P0902

### App User Interest

Overall, participants indicated considerable interest in using this app in the future. They envisioned themselves using the app daily and thought that the app would be useful in helping them manage HIV care. They stated that they would recommend the *LetSync* app to other family members, friends, and people in their social networks who are HIV positive:

I mean, all of these seem like they’re very, very helpful. I think just what it is that some of them I could see myself using day-to-day-to-day.

PID P3702

Participants liked the appearance (color and background images) of the app wireframes and felt that the app’s features and contents were appropriate. However, some thought that the app’s imagery looked somewhat provocative and said that they would not feel comfortable using the app in public spaces:

*I think the overall look is good. The only thing—this first picture?If I was in public, I wouldn’t want to click on that because it looks like it was going be a sex ad*...*Just because it’s an intimate image.* [PID P0902]

Although participants were enthusiastic about the app, they indicated a need for a more refined app presentation in terms of visual display and navigation, and they wanted the ability to personalize the aspects of the app both functionally (including tracking goals, medication adherence, and health progress) and esthetically (including adding imagery and modifying app features and colors) to make it their own. In addition, some participants commented about the amount of text on the app wireframe and suggested using less text and incorporating more icons or images instead:

Looks - I just now figured out what the silhouette was. I wasn’t even looking at the silhouette, I was looking at the foreground first for the - for the words. And text-heavy maybe? Is what I would suggest - is what I would say. Cause I didn’t - it may benefit from icons of some sort.

PID P2002

When asked about which features they were likely to use the most, most participants (20/24, 83%) expressed that they thought that all the features would be useful and commented that they look forward to using the app. They thought that they could use the app to help improve medication adherence and communication with their partners and for planning activities both for short term and long term:

Hmm, hmm, I would say all of them. I would use all of them. I may use more than others, but I would - I think all of them look pretty useful.

PID P1802

Although participants envisioned the app as being a useful resource for HIV health management*,* others thought that the app would not be helpful for them but pointed out that perhaps their partners or other people in their social networks might find it useful. Many of these individuals (22/24, 92%) already had systems in place for taking medications and setting appointment reminders, and they did not want to replace these routines:

Not for me, because if I already set a reminder and an alarm or something goes off, then I’m going to stop what I’m doing and take my meds. But for some people, that may work. With [name of index], it would probably work.

PID P4402

### Privacy and Security Concerns

Although participants were generally open about sharing health information with their partners, some felt that the decision regarding sharing health records should be entirely dependent on their and their partners’ individual preferences:

I also have a tad - I guess I am pretty transparent about all my medical issues with my partner. Yeah, I think so. I’m a little bit conditioned around your medical stuff is very private. And so - and I want him to be able to - I think it’s important for him to decide what he wants to share with me, and what he doesn’t. And vice versa. And, obviously, for us really pressing things we share with each other about - you know, I need to know all the details, unless he thinks it’s important. So that’s the part I would have to learn how to do. So I can’t really say I’d be totally into that.

PID P5101

Others had reservations about sharing detailed health information with their partners and thought that sharing of medical records in the app should be completely voluntary:

I am, intrinsically, a private person. So there are things that I don’t tell even my partner, unless it comes up. So I just think that there - everything in My Action Plan should be voluntary. Everything in Myself should be voluntarily shared.

PID P4601

When asked whether they would like pop-up reminders that they can access from the home screen on their mobile phone, participants said that they would like to receive such notifications; however, some raised concerns regarding privacy issues:

I won’t necessarily agree with that just because privacy reasons. It could pop up at the wrong time at the wrong place, and if somebody, you know - they don’t necessarily want - you know, my phone set to silent - they might not want to see, you know, have their phone at the time that pops up...Well, then if that’s the case, it’s a more discreet popup, then that’d be fine.

PID P1801

Overall, participants agreed that they would feel comfortable with using this app for HIV care management if there were security measures in place to ensure that notifications or pop-up reminders were discreet and as long as they would be able to share health information with their partners voluntarily.

## Discussion

### Principal Findings

Informed by formative studies, the *LetSync* app will be specifically designed to support individual and dyadic processes involved in optimal HIV care for partnered Black sexual minority men [5,6]. We developed the *LetSync* app wireframes to test potential features and elicit information that could be used to inform processes for designing and developing *LetSync*, an app for couples, to improve HIV care management among Black sexual minority men and their partners. In this study, we assessed the acceptability of the app wireframes among Black sexual minority male couples in 4 cities in the United States. The study provided preliminary data that helped us evaluate the potential interest in using the eventual app, elicit feedback about potential app features, and identify suggestions for app improvement. Overall, participants reported high acceptability of the app wireframes and indicated considerable interest in using the *LetSync* app in the future. In addition to near-universal interest in potential daily app use, most study participants (20/24, 83%) indicated that they would recommend the *LetSync* app to other family members, friends, and people in their social networks who are living with HIV. Our findings are consistent with those of previous studies that have shown participants’ interest in mHealth-based technologies as an acceptable resource for HIV care management and medication adherence among sexual minority male couples [11,17].

In our study, the future *LetSync* app was frequently referred to as a potential resource that could help facilitate users’ engagement in HIV care through the following mechanisms: (1) enable scheduling of appointments and timely reminders for clinic visits; (2) help improve HIV medication adherence (the app would allow them to set up reminders to take their daily doses and pick up medication refills); (3) encourage and motivate participants to ask questions and stay engaged in conversations during clinic visits; (4) facilitate effective communication by assisting couples with planning, coordination, and management of daily routines; (5) help participants understand their partner’s health needs, including access to and use of health care services; and (6) facilitate participants’ ability to improve their relationship skills, partner support, and self-efficacy in managing conflict.

In addition, study participants were enthusiastic about the app features and envisioned the future *LetSync* app to be instrumental in facilitating targeted strategies for HIV health management. Participants liked that the app would enable them to share health goals and reminders more effectively with their partners, while also including features that allowed them to maintain privacy from their partners when needed. In our previous study investigating dyadic coordination in HIV care management among Black male couples, although there were variations regarding the degree of involvement, most participants (17/24, 71%) reported being involved in their partners’ health care in terms of HIV care engagement, coordination or scheduling of clinic appointments, and providing partner support [6]. Parallel to our previous study, the findings of this study clearly indicate that designing a user-centered mHealth app with features that would enable partners to share health management goals could lead to significant improvements in coordinated HIV care among Black male couples. These findings are consistent with those of other studies that have shown the importance of couples’ engagement in everyday use of technology in preventing chronic disease progression and improving health management [18].

Examining participants’ preferences regarding the various features of the app wireframes revealed the need to ensure the development of an app that would be interactive and user centered. In our study, participants indicated that they would prefer the option to personalize the app regarding functionality, visual display, navigation, and esthetics. This finding is consistent with those of acceptability studies of apps among Black sexual minority men in the United States. For example, in a 2014 study evaluating the acceptability of an mHealth intervention to improve access to HIV prevention and care services, a key finding was the participants’ desire for customization depending on individual health needs and preferences for receiving app-related alerts and messages [17].

### Limitations

This study has several limitations. The findings obtained from the purposive sample of Black sexual minority men with HIV recruited via social networking and dating apps may not be generalizable to those who are not actively involved in these platforms. Participants in this study were recruited via web-based platforms and interviewed using internet-based methods, and therefore, generalizability of the findings may be limited to selected groups of Black sexual minority men with regular access to the internet and smartphones and those who are comfortable with the technology used. In addition, participants’ perceptions about the potential features of the *LetSync* app were based on the initial app wireframes. However, based on the information provided to participants, they were able to conceptualize its general structure and purpose, so that they could provide meaningful feedback about its acceptability and potential usefulness. Regardless of the limitations, the *LetSync* app provides an innovative approach to HIV care—an intervention that could provide Black sexual minority men and their partners with behavioral skills to effectively manage HIV and improve HIV health outcomes. In addition, insight gained from this study will be used to improve the app’s usability and likelihood of adoption among Black sexual minority men with HIV. Given that the *LetSync* app is being developed to be compatible with smartphones, the app would provide increased accessibility to users and would allow flexibility of use among Black sexual minority men and their partners throughout the United States.

### Comparison With Previous Studies

Couple-based mHealth interventions could provide an opportunity for Black sexual minority men and their partners to enhance the knowledge and skills necessary to work together to manage HIV in their relationships [19]. This study builds on formative studies that revealed interest for mobile app use among Black sexual minority men and their partners and provided insights regarding the importance of partner involvement in HIV care management in this population. By developing tailored HIV care plans using the *appointment minder* feature, enhancing problem-solving skills using *my action plan*, and cultivating HIV medication adherence using *digital pill case*, the *LetSync* app could offer a unique opportunity for couples to support each other in reaching common HIV health management goals.

Parallel to our findings, in HIV-related mHealth studies, participants expressed a preference for customizable app features [20], personalized reminders [14], motivation for medication-taking behaviors, and social support connection or networking features [21]. Although mHealth interventions including SMS text messages, emails, and social media platforms have been shown to improve HIV prevention and treatment among sexual minority men, they primarily used one-way messaging approaches, which often lacked the interactive and personalization features needed for delivering care that is tailored to each participant’s specific health needs and HIV management goals [15,22].

In addition, all the reviewed mHealth HIV intervention studies were limited in scope, as they focused mainly on individual-centered interventions, with none of them focusing on developing an mHealth app aimed at improving HIV care engagement and treatment among partnered Black sexual minority men in the United States. Our study focuses on developing a couple-centered mobile app that addresses multiple aspects along the HIV continuum of care, including facilitating engagement and retention in HIV care for partnered individuals, improving medication adherence, and strengthening problem-solving skills to improve HIV health management goals among partnered Black sexual minority men.

### Conclusions

Our findings revealed considerable interest in app use among Black sexual minority men in couples, which would possibly increase the chance of the *LetSync* app being successfully adopted by participants. In addition, the study findings revealed specific areas of the app wireframes that need improvement—information that will help inform the development of app features that Black men would find as helpful and appropriate to use (with their partners) for HIV care management. For Black men who struggle with poor communication with partners or providers, the *LetSync* app could help increase ease in communicating health needs to their partners and providers and could provide an acceptable couple-centered modality for these men to receive support in accessing HIV care services. During interviews, participants viewed the *appointment minder* feature as a potential resource to help improve transparency between couples. They felt that this feature could allow easy sharing of health information and planning of clinic visits and daily activities. In addition, participants thought that the *LetSync* app could help prevent schedule-related conflicts and unnecessary arguments between couples. Participants were also enthusiastic about the feature’s potential to help couples build trust and foster strong relationships. They also thought that *appointment minder* would enable them to better understand their partners’ health needs and could help them formulate targeted strategies to provide physical, mental, and emotional support tailored to their partners, particularly during stressful medical situations.

## Appendix

Multimedia Appendix 1 In-depth interview guide.

Multimedia Appendix 2 Screenshots of the LetSync app wireframes.

## Abbreviations

mHealth: mobile health
PID: participant ID
