# Supplementary material for: Acceptability of the LetSync App Wireframes for an mHealth Intervention to Improve HIV Care Engagement and Treatment Among Black Partnered Sexual Minority Men: Findings from In-Depth Qualitative Interviews
Source: JMIR Form Res. 2023 Aug 25;7:e43676. doi: 10.2196/43676 (PMC10492169; doi:10.2196/43676)
Supplement: Multimedia Appendix 1 [file formative_v7i1e43676_app1.pdf]

In-depth Interview Guide
LetSync App Wireframes Feedback

A. Invision Mock-Up

Open mock-ups on Invision. Display the Main Menu and share screen on Zoom.

1. First, let me show you the Main Menu. This is what you first see when you open the app.
   i. What do you think about the appearance? What do you think about how it looks?
   ii. What do you think of the overall look? This whole screen, the text, picture, color, everything.

2. Next, I'll go through the different features on here and explain what they do.

Go through each feature (My Action Plan, Myself, My Partner, Surveys…etc.) and give a brief overview of them. Answer any questions that the participant has about the features.

3. We just went through the main features of the app wireframes. What do you think about everything so far? That overall set up?
   - ➔ What are your initial impressions of this app, from everything - the picture in the background, the text, the color, the layout, everything?
     1. Could you tell me what features do you like right off the bat?
     2. What would you change about this feature?
   - ➔ What features do you feel "meh" about?
     1. What would you change about this feature? Or something that you would add or remove from this feature/app?
   - ➔ What features do you not like?
     1. Is there anything specific that you don't like about it?
     2. What would you change about this feature?
4. What features can you see yourself or your partner using a lot?
5. What features do you think you or your partner wouldn't use as much?
6. Would you find this app useful? And would you recommend it?

B. **Other Features**

1. Thanks for sharing your thoughts on the mock-up! Now, we want to get your thoughts on several features that aren't on the mock-up right now, but that we are thinking of adding.

    i. What if there was a button that you could press that changes colors, to help you remember to take your meds? Would you find this useful?
        1. What if the button popped up over other apps? How useful would this be?

    ii. How about a feature that lets you keep track of appointments you have, and gives you suggestions on what to ask the doctor based on the type of appointment?

    iii. What do you think about splash screens that contain quotes of encouragement?

C. **Closing and thank you.**

1. Are there any other thoughts you have about the app or what you would want to see on it?
2. *Proceed to conclude the interview.*
