# Supplementary material for: Acceptability of the LetSync App Wireframes for an mHealth Intervention to Improve HIV Care Engagement and Treatment Among Black Partnered Sexual Minority Men: Findings from In-Depth Qualitative Interviews
Source: JMIR Form Res. 2023 Aug 25;7:e43676. doi: 10.2196/43676 (PMC10492169; doi:10.2196/43676)

Multimedia Appendix 2: Screenshots of the LetSync app wireframes

Snapshots of Appointment Minder

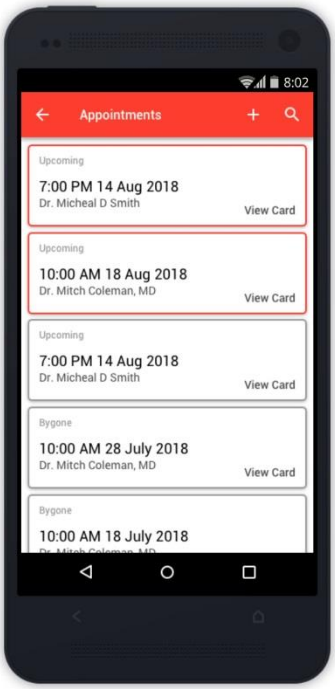

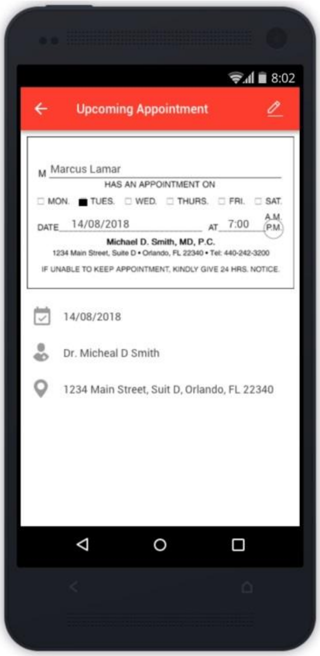

Snapshots of My Action Plan

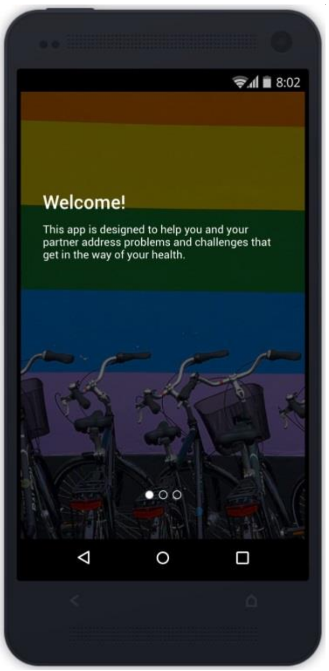

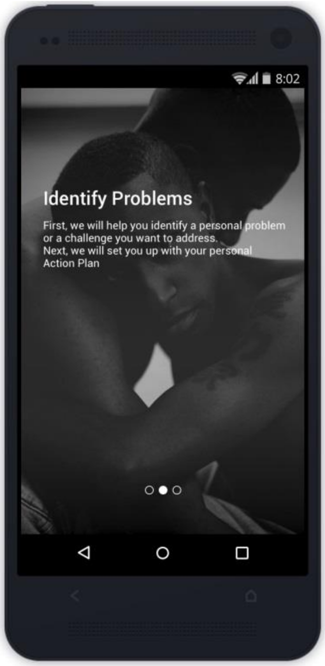

Supplement: Multimedia Appendix 2 [file formative_v7i1e43676_app2.pdf]
